# An Atypical Pathogen at an Atypical Location: A Rare Case of Salmonella-Associated Submandibular Abscess

**DOI:** 10.7759/cureus.66026

**Published:** 2024-08-02

**Authors:** Lavanya Balaji, Harish Manoharan, Neelusree Prabhakaran

**Affiliations:** 1 Department of Microbiology, Saveetha Medical College and Hospitals, Saveetha Institute of Medical and Technical Sciences, Saveetha University, Chennai, IND

**Keywords:** extra-intestinal salmonellosis, incision and drainage of abscess, salmonella paratyphi b, submandibular abscess, atypical salmonella

## Abstract

*Salmonella* infections commonly cause gastroenteritis and enteric fever but can also result in extraintestinal infections, especially in immunocompromised individuals. Although rare, *Salmonella* infection in the head and neck region was found to be more common in diabetics and patients with malignancy. We present a unique case of a 52-year-old immunocompromised man with uncontrolled diabetes who developed a submandibular abscess due to *Salmonella* Paratyphi B. The patient initially presented with pain, swelling, and difficulty swallowing, which worsened over a week. Diagnostic imaging revealed a well-defined abscess with lymphadenopathy. Management included incision and drainage, followed by identification of *Salmonella* Paratyphi B and targeted antibiotic therapy based on culture and sensitivity analysis. This case highlights the importance of early recognition, appropriate diagnostic imaging, and targeted antimicrobial therapy in managing uncommon manifestations of *Salmonella* infection in the head and neck. Continued vigilance and multidisciplinary management are essential for favorable patient outcomes in such cases.

## Introduction

*Salmonella*, a non-encapsulated Gram-negative motile bacillus, commonly causes typhoid fever, enterocolitis, or bacteremia with focal lesions [1]. Enteric fever, caused by *Salmonella* typhi, *Salmonella* Paratyphi A, and *Salmonella* Paratyphi B, is prevalent in developing tropical countries but increasingly seen in developed countries as an imported infection. Typhoid fever is estimated to affect 11-21 million people worldwide each year, leading to 120,000-160,000 deaths. In India, for instance, the Global Burden of Disease Study in 2017 reported an incidence of 586 cases per 100,000 person-years for typhoid/paratyphoid infections, highlighting a significant burden in developing regions compared to developed countries [2]. *Salmonella* infections encompass a spectrum of clinical manifestations, primarily known for their gastrointestinal repercussions, including gastroenteritis and enteric fever. However, in immunocompromised individuals or those with significant underlying conditions such as HIV, diabetes, or malignancy, *Salmonella* can lead to focal infections affecting various organs beyond the gastrointestinal tract [3]. These extraintestinal manifestations are often secondary to bacteremia or lymphatic dissemination and involve diverse anatomical sites. The development of a neck abscess due to *Salmonella* typically follows bacteremia, where the bacteria enter the bloodstream and disseminate to distant sites. This is particularly likely in individuals with compromised immune systems or pre-existing conditions predisposing them to bacterial spread. Soft-tissue infections by *S*. typhi originate from hematogenous or lymphatic spread of gastrointestinal infections. *Salmonella *can spread via gastrointestinal lymphatics or through oropharyngeal tonsil tissue. Neck vulnerability to bacterial seeding is influenced by lymphatic drainage, proximity to respiratory and digestive tracts, and immune responses impacting localized infection outcomes such as abscesses or lymphadenitis [4]. The body’s immune response to the infection results in the accumulation of white blood cells, dead tissue, and bacteria, which form pus. While focal salmonellosis involving the head and neck region is rare, documented cases exist, particularly among immunocompromised individuals and pediatric patients [5-7]. Most commonly associated with bacterial parotitis [8], pathogens like *Staphylococcus aureus* dominate clinical presentations, although *Salmonella* has been sporadically reported [9]. Notably, only a few isolated cases of *Salmonella* infection involving the submandibular gland have previously been documented in medical literature [9,10]. Complications include respiratory obstruction due to massive swelling of the neck, infection of deep spaces of the head and neck, fistula formation, osteomyelitis of adjacent bones, septic jugular thrombophlebitis, septicemia, and meningitis [6]. Deep neck infections require timely diagnosis and treatment. Diagnosis involves clinical examination and imaging studies like ultrasound, computed tomography scan, or magnetic resonance imaging. Laboratory tests, such as blood cultures and pus cultures, are crucial for identifying the causative organism. Managing a neck abscess caused by *Salmonella* involves surgical drainage and antibiotic therapy tailored to the antibiotic sensitivity profile of the isolated strain. Commonly used antibiotics include fluoroquinolones, third-generation cephalosporins, and ampicillin. In severe cases or immunocompromised patients, a prolonged course of antibiotics may be necessary [11]. Here, we present a unique case report detailing a *Salmonella* infection manifesting as a neck abscess involving the submandibular gland. This report aims to provide a comprehensive review of existing literature on *Salmonella* infections of the head and neck, highlighting the clinical presentation, diagnosis, management, and outcomes associated with this uncommon yet noteworthy condition.

## Case presentation

A 52-year-old immunocompromised man presented to the outpatient department with a complaint of pain and swelling over the right submandibular region. The patient presented with a week's history of pain and swelling in the right submandibular region, worsening over the last two days, with uncontrollable pain and difficulty swallowing. The patient is a known case of type 2 diabetes mellitus for 10 years on irregular medication. The patient is a non-smoker and non-alcoholic. On physical examination, the patient was febrile (100.4℉) with stable vitals (blood pressure was 110/80 mmHg, respiratory rate, 20 breaths/min, oxygen saturation, 99% on room air and pulse rate, 84 beats/ minute). Assessment of the oral cavity revealed poor hygiene, evidenced by plaque accumulation and gingival recession; however, no dental caries was observed. On local examination, the swelling was 5×6 cm and firm in consistency, with a smooth surface on the right submandibular region. It was warm and tender to the touch, and the right lymph nodes were palpable at Level Ia and Ib. Routine laboratory investigations (Table 1) revealed elevated white blood cell count and elevated C-reactive protein and erythrocyte sedimentation rate, suggestive of an ongoing infection.

**Table 1:** Routine laboratory investigations of the patient with reference ranges.

Parameter	Patient Value	Reference Values
White blood cell count	14870 cells/cumm	40-10000 cells/cumm
Neutrophils	75.5%	44.0-72.0%
Lymphocytes	18.1%	18.0-59.0%
Monocytes	2.0%	0.0-12.0%
Eosinophils	0.0%	0.0-10.0%
Basophils	0.1%	0.0-3.0%
Red blood cell count	5.17 million/cumm	3.76-5.50 million/cumm
Hemoglobin	14.4 g/dL	11.3-15.2 g/dL
Hematocrit	38.5%	33.4-44.9%
Platelet count	3.13 lakhs/cumm	1.5-4.1 lakhs/cumm
Absolute neutrophil count	11180 cells/cumm	2000-7000 cells/cumm
Aspartate aminotransferase (ALT)	41 IU/L	5-50 IU/L
Alanine aminotransferase (AST)	31 IU/L	17-59 IU/L
Alkaline phosphatase level (AKP)	106 IU/L	38-126 U/L
Albumin	4.8 g/dl	3.5-5.0 g/dl
Creatinine level	0.60 mg/dL	0.40- 1.10 mg/dL
C-reactive protein level	80 mg/L	<10 mg/L
HbA1C	10.3%	<6 %
Random Blood Glucose Level	226 mg/dl	80-40 mg/dl
Erythrocyte sedimentation level (mm/hour)	120 mm/hr	0-14 mm/hr

**Figure 1:**
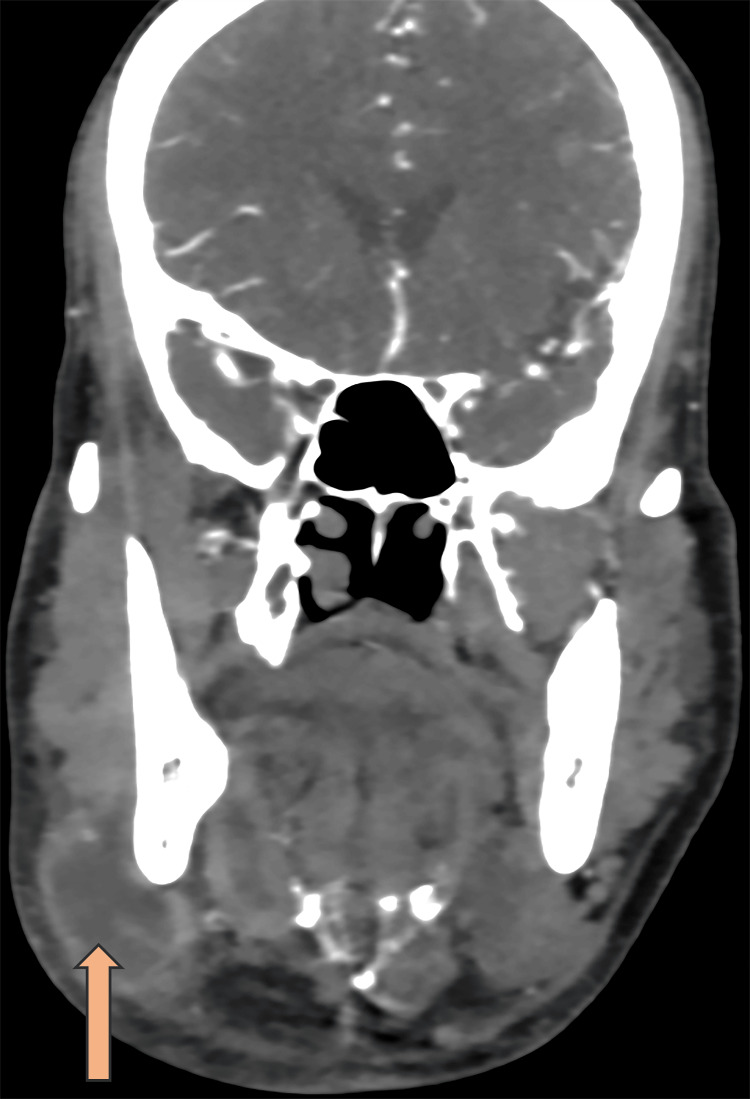
Coronal-section contrast-enhanced computed tomography of the right submandibular region. Coronal-section contrast-enhanced computed tomography revealing a thick-walled, well-defined, differentially dense lesion/necrotic nodal mass in the right submandibular region.

Radiological imaging like ultrasonography (USG) and contrast-enhanced computed tomography (CECT) of the right submandibular region revealed a well-defined, thick-walled cyst with smooth margins with internal contents showing varying densities: higher attenuation (pus) surrounded by lower attenuation areas (necrotic material). Gas formation, indicated by septations or air pockets, is strongly indicative of an infective etiology conclusive of submandibular abscess with lymphadenopathy (Figure 1). Upon initial evaluation, the patient was provisionally diagnosed with a submandibular abscess and was admitted for further assessment and a potential Incision and Drainage (I&D) procedure.

**Figure 2:**
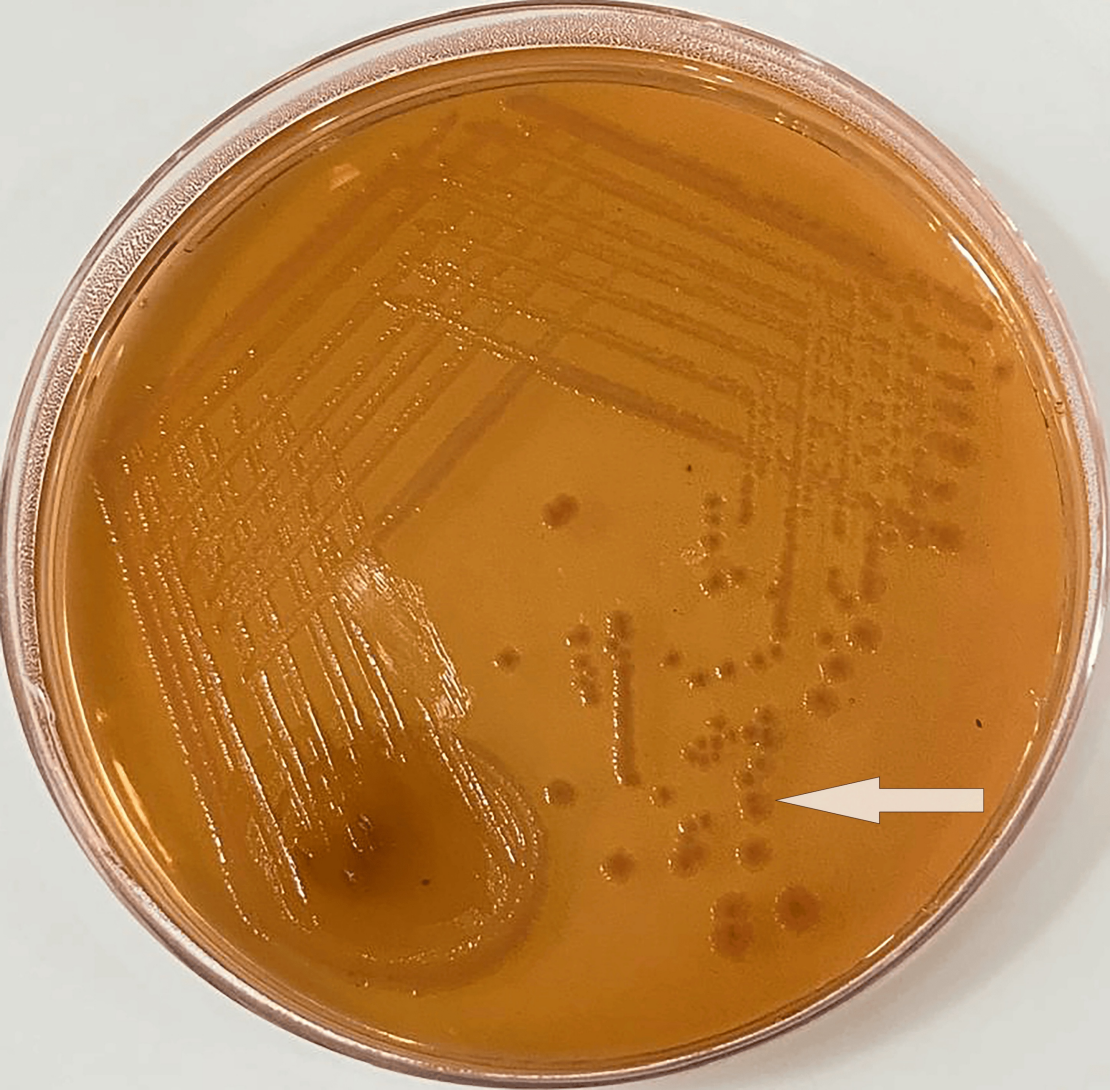
MacConkey agar showing non-lactose fermenting colonies.

The elevated HbA1C and random blood glucose levels indicate poorly controlled diabetes (Table 1), necessitating immediate attention to glycemic management. Serological tests for HIV, HbsAg, and HCV returned negative results. Once glycemic control was achieved, the I&D procedure was performed under local anesthesia, with the aspirate being sent to the microbiological laboratory for Acid fast staining, culture, and sensitivity analysis. The wound dressing was changed every other day, and the patient was empirically started on parenteral amoxicillin/clavulanic acid 1.2g twice daily and parenteral metronidazole 500 mg twice daily. The acid-fast staining with 25% sulfuric acid showed the absence of acid-fast bacilli, ruling out the tuberculous abscess. Gram stain of the sample revealed plenty of pus cells with gram-negative bacilli. The sample was sub-cultured on blood, chocolate, and MacConkey agar. After 24 hours of incubation aerobically at 37℃, blood agar and chocolate agar showed moderately large 2-3mm in diameter, grey, white, moist, circular disc with a smooth convex surface and entire edges, while MacConkey agar showed non-lactose fermenting colonies of size 1-3 mm in diameter (Figure 2). The organism was identified as *Salmonella* Para typhi B using the Vitek2 Compact system (for automated identification and antibiotic susceptibility testing) by BIOMERIEUX. Serotyping was done via the sero-agglutination test using anti-sera against O and H antigens of *S*. Typhi and *S*. Paratyphi A and B.

Based on the antibiotic susceptibility testing by disk diffusion on Mueller-Hinton agar, it was determined that the isolate was susceptible to ampicillin, ceftriaxone, cotrimoxazole, tetracycline, levofloxacin, and azithromycin according to Clinical and Laboratory Standards Institute, 2022 guidelines. Blood culture sets were obtained which showed no growth after seven days and urine culture showed no bacteriuria. In light of the positive culture, the patient was introduced to parenteral ceftriaxone 1g twice daily and oral azithromycin 500mg once daily for two weeks. Upon symptomatic improvement, the patient was changed to oral cefixime 200mg twice daily and oral azithromycin 500mg once daily and discharged. The patient was advised to follow up with the outpatient department. On follow-up, the wound had healed without complications, and the blood glucose levels were under control.

## Discussion

Neck abscesses due to *Salmonella* infection are rare but notable manifestations, particularly in individuals with predisposing systemic conditions such as diabetes mellitus or malignancies [12]. These conditions can compromise the immune system, increasing susceptibility to *Salmonella *species and potentially leading to severe infections. Diabetes mellitus, particularly when uncontrolled, can impair several aspects of the immune response, including neutrophil function, humoral immunity, and cell-mediated immunity. This immunocompromised state can make it difficult to clear infections, contributing to the severity and atypical nature of infections like those caused by *Salmonella* species [13]. The elevated HbA1C and random blood glucose levels in this patient underscore the importance of glycemic control in managing and preventing infections. Case reports by McLeod et al. [12] and Pastagia et al. [13] align with our findings. *Salmonella* infections in the head and neck region are typically polymicrobial, involving pathogens like *Streptococcus viridans, Staphylococcus aureus, Klebsiella pneumoniae*, and anaerobic bacteria such as *Bacteroides, Peptostreptococcus, Fusobacterium, and Prevotella* [14]. However, Salmonella infections in the head and neck may differ from other bacterial infections, necessitating early and accurate diagnosis through imaging studies like USG and CECT, which are crucial in identifying abscess formation and guiding treatment. In this case, imaging revealed a well-defined, differentially dense lesion/necrotic nodal mass in the right submandibular region, confirming an infective etiology and ruling out malignancy [15,16].

Management of invasive *Salmonella* infections, including neck abscesses, requires prompt initiation of appropriate antibiotics within 72 hours. Empiric therapy often includes beta-lactam/beta-lactamase inhibitors, third-generation cephalosporins like ceftriaxone or cefixime, or fluoroquinolones such as ciprofloxacin or levofloxacin. In this case, the patient was initially treated with parenteral amoxicillin/clavulanic acid 1.2g and metronidazole 500mg twice daily. Upon identification of* Salmonella* Para typhi B and based on antibiotic susceptibility testing, the treatment regimen was adjusted to parenteral Ceftriaxone 1g twice daily and oral azithromycin 500mg once daily, leading to clinical improvement [17,18]. Surgical intervention with incision and drainage may be necessary, particularly in cases where abscess formation is evident or when the infection does not respond adequately to antibiotics alone, as seen in our patient. Surgical exploration allows for microbiological cultures to guide targeted antibiotic therapy, considering antibiotic resistance patterns [19]. The duration of antimicrobial therapy varies, typically spanning two weeks for neck abscesses, with longer durations (four to six weeks) for other sites to ensure complete eradication and prevent recurrence [20].

## Conclusions

This case report describes a rare instance of *Salmonella* infection presenting as a submandibular abscess in an immunocompromised patient with uncontrolled diabetes mellitus. It also underscores the complexity of *Salmonella* infections beyond the gastrointestinal context and the need to consider unusual pathogens in patients with systemic conditions, particularly in the head and neck. A multidisciplinary approach was crucial, beginning with clinical and radiological suspicion, followed by surgical intervention and microbiological analysis confirming *Salmonella* Paratyphi B. Targeted antibiotic therapy, based on susceptibility testing, led to clinical improvement and abscess resolution. This case underscores the importance of early diagnosis, targeted antimicrobial therapy, and adherence to antimicrobial stewardship in managing uncommon bacterial infections. Continued vigilance and clinical awareness are essential to promptly recognize and manage similar cases, thereby improving patient outcomes and reducing the burden of severe bacterial infections in vulnerable populations.
